# Asymmetric Notch activity by differential inheritance of lysosomes in human neural stem cells

**DOI:** 10.1126/sciadv.abl5792

**Published:** 2022-02-11

**Authors:** Bettina Bohl, Ammar Jabali, Julia Ladewig, Philipp Koch

**Affiliations:** 1 Department of Translational Brain Research, Central Institute of Mental Health, University of Heidelberg/Medical Faculty Mannheim, Mannheim, Germany.; 2 HITBR Hector Institute for Translational Brain Research gGmbH, Mannheim, Germany.; 3 German Cancer Research Center (DKFZ) , Heidelberg, Germany.

## Abstract

Symmetric and asymmetric cell divisions are conserved strategies for stem cell expansion and the generation of more committed progeny, respectively. Here, we demonstrate that in human neural stem cells (NSCs), lysosomes are asymmetrically inherited during mitosis. We show that lysosomes contain Notch receptors and that Notch activation occurs the acidic lysosome environment. The lysosome asymmetry correlates with the expression of the Notch target gene *HES1* and the activity of Notch signaling in the daughter cells. Furthermore, an asymmetry of lysosomes and Notch receptors was also observed in a human organoid model of brain development with mitotic figures showing preferential inheritance of lysosomes and Notch receptor in that daughter cell remaining attached to the apical membrane. Thus, this study suggests a previously unknown function of lysosomes as a signaling hub to establish a bias in Notch signaling activity between daughter cells after an asymmetric cell division of human NSCs.

## Teaser

Inheritance of lysosomes in dividing neural stem cells correlates with Notch activity and positional identity in brain organoids.

## INTRODUCTION

Stem cells are defined by both their ability to self-renew and their ability to produce cells that differentiate into more specified derivatives. Asymmetric cell division—the generation of two distinct siblings during mitosis—is a mechanism to generate more committed cells and cellular diversity. In cells undergoing an asymmetric division, cell fate determinants are asymmetrically inherited by the two daughter cells—a process referred to as intrinsic asymmetry. Applying different model organisms and cellular lineages, multiple such determinants have been identified including mRNAs, proteins, protein complexes, aggregates, and organelles such as components of the endoplasmic reticulum, mitochondria, and vesicular structures [reviewed in (*1*–*3*)]. The latter include a subset of endosomes and, as recently demonstrated in the hematopoietic lineage, lysosomes (*3*). More specifically, in sensory organ precursors (SOPs) of *Drosophila melanogaster* as well as the zebrafish spinal cord, a subset of endosomes marked by the protein Smad anchor for receptor activation (Sara) has been demonstrated to be preferentially inherited by the pIIa and the p cells, respectively, and that Sara endosomes contain Notch receptors and ligands (*4*, *5*). A very recent study demonstrated a similar mechanism in the zebrafish forebrain, where Notch ligands are asymmetrically distributed after internalization into endosomes (*6*). In the human hematopoietic lineage, lysosomes, autophagosomes, and mitophagosomes have been shown to segregate asymmetrically in some daughter cells, and this is associated with a different metabolic and oxidative state of the receiving cells (*3*). It was suggested but not demonstrated that Notch signaling might also play a role in this system. Here, we set out to illuminate whether subcellular vesicular components of the endolysosomal system are asymmetrically inherited by dividing human neural stem cells (NSCs) generated from induced pluripotent stem cells (iPSCs). We demonstrate that lysosomes segregate asymmetrically in a subset of dividing NSCs. We further demonstrate that ligand-bound Notch receptor shuffle through the endocytic pathway into lysosomes and that the receiving cells show differences in Notch signal activity depending on lysosome inheritance. Last, we demonstrate that an asymmetry of lysosomes and Notch is also present in asymmetrically dividing apical radial glia cells in an organoid model of human brain development.

## RESULTS

### Human iPSCs-derived NSCs to study cellular asymmetry

**Fig. 1.**
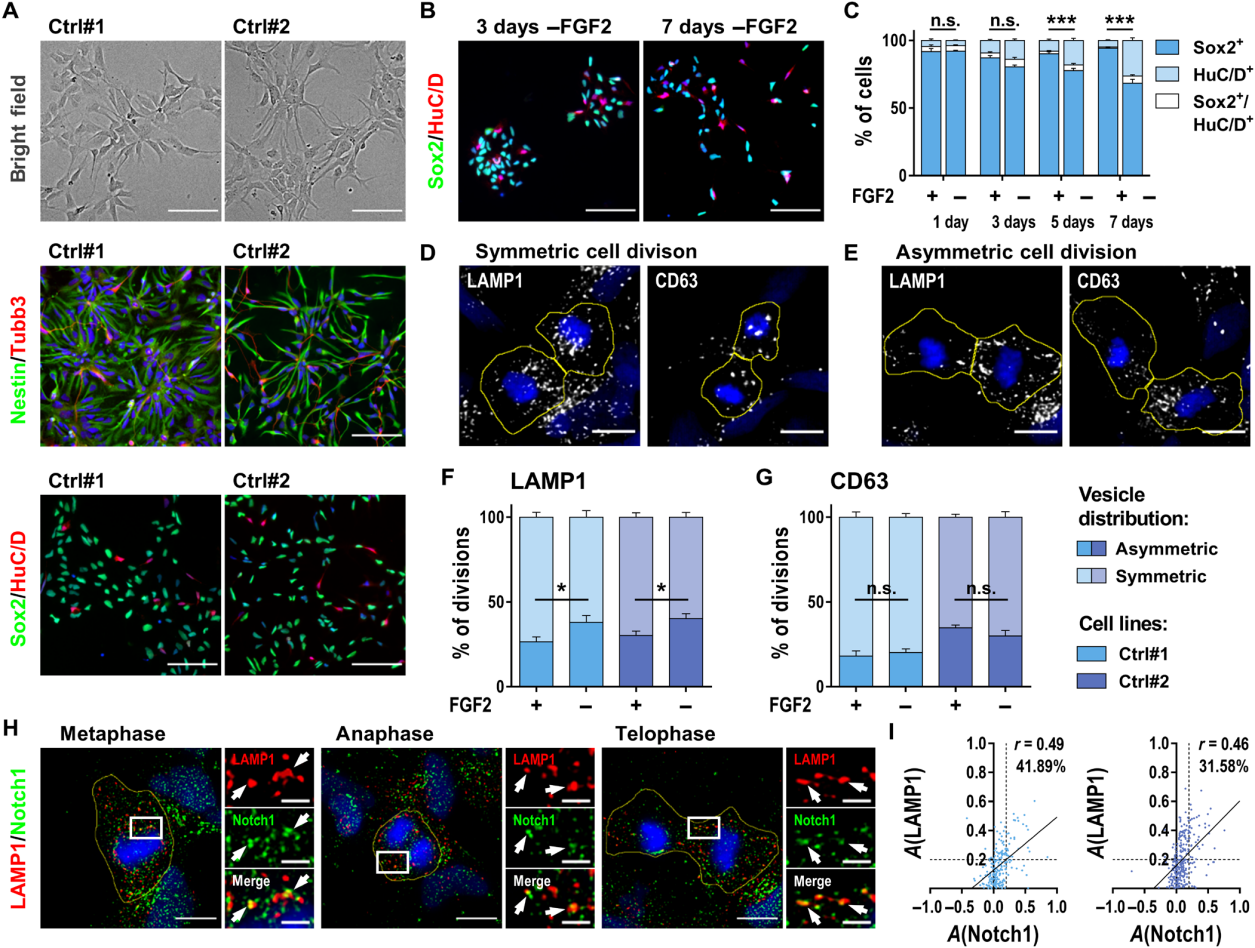
Human NSCs tend to differentiation upon FGF2 withdrawal accompanied by an increase in asymmetric LAMP1 segregation during mitosis. (**A**) Representative brightfield images and immunostaining of rosette-type NSCs from two healthy control cell lines (Ctrl#1 and Ctrl#2) for NSC markers (Sox2 and Nestin) and neuron markers (Tubb3 and HuC/D). (**B** and **C**) Immunostaining for Sox2 and HuC/D and respective quantification of the amount of NSCs (Sox2^+^) and neurons (HuC/D^+^) after FGF2 withdrawal from Ctrl#1-NSCs over a time course of 7 days (*n* = 3, two-way ANOVA with Bonferroni’s multiple comparison test, means + SEM). (**D** and **E**) Representative images of symmetric and asymmetric segregation of LAMP1^+^ and CD63^+^ vesicles during mitosis of Ctrl#1-NSCs. Yellow lines indicate ROIs surrounding daughter cells, defined by DAPI and phalloidin staining (see also fig. S1E). (**F** and **G**) Quantification of LAMP1 and CD63 asymmetry based on sum intensity ratios between paired daughter cells (see fig. S1D) in conditions with and without FGF2 (*N* = 6 coverslips from three independent experiments with *n* = 42 to 58 cells per coverslip, two-way ANOVA with Bonferroni’s multiple comparison test, means + SEM). (**H**) Representative images of LAMP1 costained with Notch1 receptors in different mitotic phases of Ctrl#1-NSCs. (**I**) Correlation of asymmetry indices *A* of LAMP1 and Notch1 in Ctrl#1 and Ctrl#2-NSCs. Dots represent individual mitotic event, and linear regression is shown. Scale bars, 100 μm (A and B), 10 μm (D, E, and H), 5 μm [zoomed in (H)]. DNA was counterstained with DAPI. *P < 0.05 and ***P < 0.001. n.s., not significant. See also fig. S1.

As an experimental model to investigate symmetric or asymmetric inheritance of subcellular components, we used a stably expandable NSC population derived from human iPSCs [fig. S1A and (*7*)]. These cells can be expanded in the presence of the growth factors fibroblast growth factor 2 (FGF2) and epithelial growth factor (EGF) without changing their growth kinetics or differentiation potential upon growth factor withdrawal (*7*, *8*). A characteristic feature of these NSCs is that they form rosette-like structures in vitro. Figure 1A shows the typical growth pattern of the two cell lines applied in this study and generated from two independent healthy individuals. Rosettes generated by the cells show an apical-basal polarity with the central lumen marked by the tight junction marker zonula occludens-1 and the polarity factor protein kinase C λ (fig. S1B). In the presence of growth factors, most of the cells stain positive for the NSC-associated transcription factor Sox2 and intermediate filament Nestin with only few cells expressing neuronal markers such as the human neuronal protein HuC/D or β III tubulin (Tubb3) (Fig. 1A). The stable proliferation of the cultures argues for a largely symmetric mode of cell division under these conditions. The low percentage number of occurring neurons also under these expanding conditions further indicates spontaneous differentiation of a smaller fraction of the cells. We next investigated the dynamics of differentiation upon FGF2 withdrawal by investigating Sox2 and HuC/D over the time course of 1 week. Over the 7 days analyzed, total cell numbers remained largely stable (fig. S1C). After 3 days, we observed a slight increase of HuC/D^+^ cells (14.0 ± 1.2% of the total cell number). By 5 days of differentiation, the percentage of HuC/D^+^ neurons had increased to 18.1 ± 1.6% and reached 26.2 ± 2.0% at 7 days of differentiation (Fig. 1C). Extrapolating from these differentiation dynamics, we choose day 3 after FGF2 withdrawal as the time point to investigate symmetric and/or asymmetric inheritance of subcellular vesicular components in further experiments as this time point precedes significant changes in the cellular composition of the cultures.

### Asymmetric inheritance of lysosomes correlates with asymmetric distribution of Notch receptors in dividing NSCs

We then looked for the distribution of antigens marking different components of the endolysosomal compartment and quantified their symmetric or asymmetric inheritance. To this end, we established an asymmetry index (*A*) calculated from the intensity sums of immunofluorescence staining in paired daughter cells during telophase of mitosis (fig. S1D). The mitotic phase and the area of the daughter cells were determined on the basis of DNA and actin staining, respectively (fig. S1E), and a mitotic event was considered asymmetric with *A* > 0.2. This corresponds to a 50% higher sum intensity in one of the daughter cells compared to the sibling. We found that in a small percentage of divisions, the early endosome markers early endosome antigen 1 (EEA1) and Rab5 were asymmetrically segregated, whereas late endosomes (Rab7^+^), recycling endosomes (Rab11^+^), the subpopulation of Sara^+^ endosomes, and autophagosomes (LC3^+^) were very rarely distributed asymmetrically (fig. S1F). In a significant number of mitotic events analyzed, lysosomes marked by lysosomal-associated membrane protein 1 (LAMP1) and LAMP2 as well as multivesicular bodies marked by CD63 were asymmetrically segregated into the two daughter cells (Fig. 1, D and E, and fig. S1F). This result was confirmed when analyzing the number of vesicles segregated between daughter cells (fig. S1G). To connect this observation with a decision over the cell fate of the daughter cells, we directly compared the asymmetry of LAMP1 and CD63 in proliferating cultures (+FGF2) and differentiating cultures (−FGF2). We found a significant increase in asymmetrically segregated LAMP1 from 26.53 ± 2.84% and 30.21 ± 2.62% to 38.05 ± 3.92% and 40.21 ± 2.83% in Ctrl#1 and Ctrl#2, respectively, whereas the percentage of asymmetric distribution of CD63 was not changed upon FGF2 withdrawal (Fig. 1, F and G). These results suggest a directed segregation of LAMP1^+^ vesicles during mitosis of NSCs cultured in differentiation-promoting conditions. The percentage of asymmetrically dividing cells seemed highly conserved between the two independent NSC lines, suggesting a common underlying mechanism facilitating the switch from proliferative to neurogenic cell division.

We next got interested whether cell fate determinants inside of the LAMP1^+^ vesicles could be identified. Asymmetric segregation of Notch signaling components has been described as a mediator of asymmetric cell divisions and cellular fate determinants in NSCs of several organisms (*4*, *6*, *9*, *10*). We thus investigated Notch1 and its subcellular distribution. Notch1 staining using different antibodies resulted in a Notch1 distribution with a vesicular pattern, which is in line with the idea that Notch1, upon binding of its ligand, is processed via endolysosomal trafficking [fig. S1H and (*11*–*13*)]. We then looked for colocalization of Notch1 with LAMP1 and found overlap in several of the stained vesicular structures throughout the mitotic cycle (investigated in metaphase, anaphase, and telophase; Fig. 1H). A co-occurrence of Notch1 with CD63^+^ vesicles was also detectable but significantly smaller (fig. S2, A and B). The overall distribution of Notch1 receptors between daughter cells was quantified similar to LAMP1 and showed a positive correlation between Notch1 receptor and LAMP1 distribution within each daughter cell pair. In 41.89 and 31.58% of the cell divisions analyzed in Ctrl#1 and Ctrl#2, respectively, Notch1 and LAMP1 are asymmetrically coinherited by the LAMP1^high^ daughter cell (Fig. 1I), whereas no mitotic events with asymmetric inheritance in opposing daughter cells were found. This correlation let us hypothesize that LAMP1 asymmetry might influences the cell fate decision, at least in part, by biasing Notch signaling activity toward one of the daughter cells.

### Ligand-bound Notch1 receptors are endocytosed via clathrin-mediated endocytosis and transported to lysosomes

**Fig. 2.**
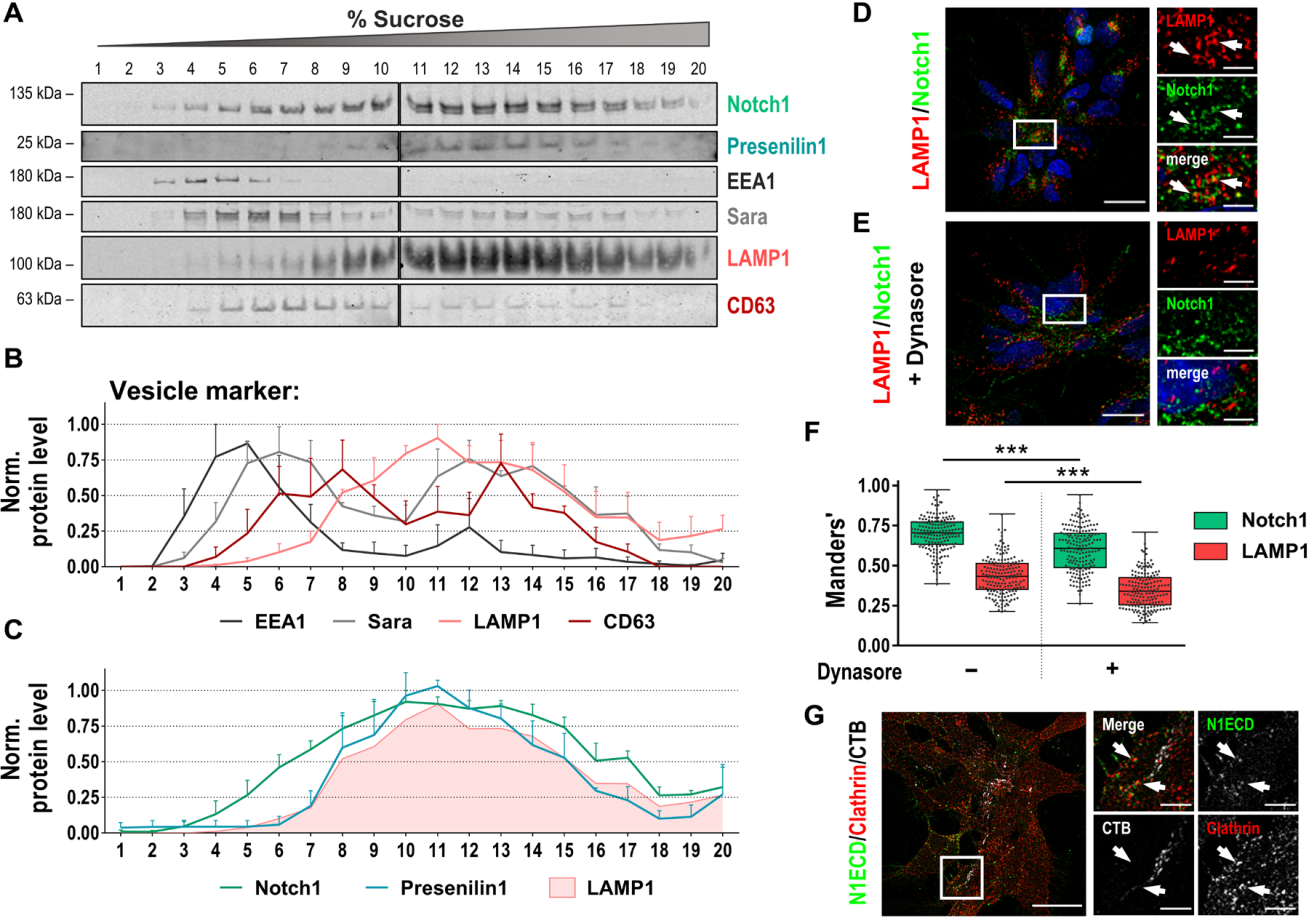
Notch1 receptors are accumulated in lysosomes after clathrin-mediated endocytosis. (**A**) Representative Western blot of sucrose gradient fractions collected from Ctrl#1-NSCs. (**B** and **C**) Protein quantification of vesicle markers (B), Notch1 receptors and γ-secretase subunit Presenilin1 (C) along the sucrose gradient [*n* = 3, means + SEM, mean of LAMP1 again in (C) as reference]. (**D** and **E**) Immunostaining for LAMP1 and Notch1 in Ctrl#2-NSCs treated for 1 hour with 0.1% (v/v) DMSO (D) or 50 μM dynasore (E). (**F**) Manders’ co-occurrence analysis of LAMP1 and Notch1 receptors with and without dynasore treatment (*N* = 179 cells from three independent experiments, Kruskal-Wallis test with Dunn’s multiple comparison test, box plot with dots for individual cells). (**G**) Representative images of Ctrl#2-NSCs stained with CTB-AF555 and immunostained for the N1ECD and clathrin. Scale bars, 20 μm (D, E, and G) and 5 μm [zoomed in (D), (E), and (G)]. Nuclei were counterstained with DAPI in (D and E). ***P < 0.001.

The immunocytochemical analysis of the localization of Notch1 and LAMP1 in NSCs suggests the receptors to be present within LAMP1^+^ lysosomes. To further validate this finding, subcellular components were separated using sucrose gradient centrifugation (*14*). When investigating the presence of Notch1 and LAMP1 in the different fractions of the gradient, both antigens were detected in a highly similar pattern, indicating the presence of both antigens in the same subcellular compartments (Fig. 2, A to C). In contrast, other vesicle markers displayed different profiles within the fractions. CD63^+^ particles and the Sara^+^ subpopulation of endosomes still showed a partial overlap with Notch1 receptors but a stronger enrichment in low-sucrose fractions, where the endosomal marker EEA1 prominently accumulated. This indicates that in our cellular system, Notch1 receptors are not residing within (early) endosomes but are transported toward lysosomes. Notably, Presenilin1 as a major component of the γ-secretase complex showed a distribution pattern similar to Notch1 and LAMP1, which might indicate that S3 cleavage of Notch1 by the protease complex occurs in lysosomes.

**Fig. 3.**
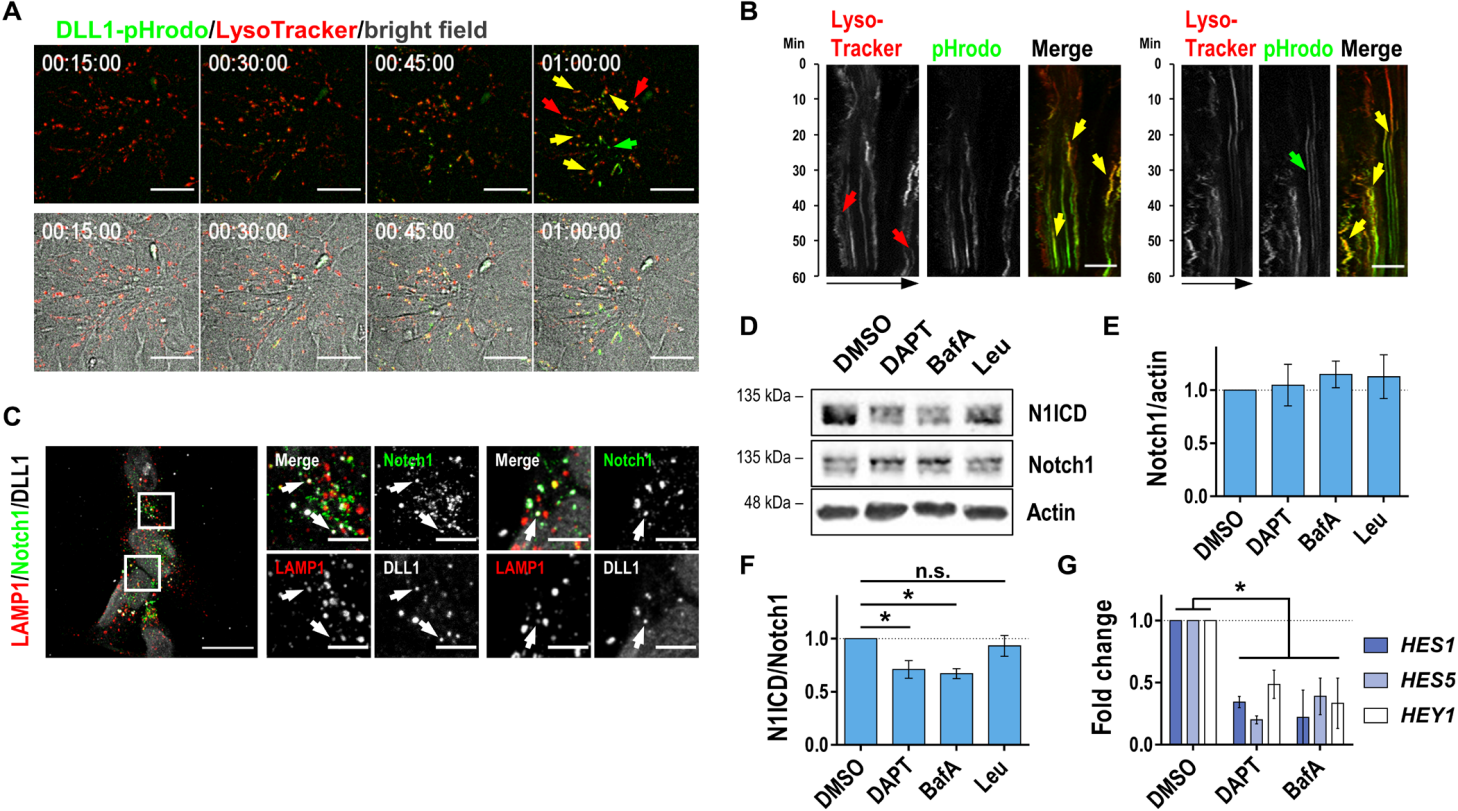
Notch internalization is triggered by ligand binding and vesicle acidification is necessary for receptor activation. (**A**) Representative frames from live-cell imaging of LysoTracker-stained Ctrl#2-NSCs starting after addition of DLL1-pHrodo at time point 0. LysoTracker- and pHrodo single-positive vesicular structures are indicated by red and green arrows, respectively, double-positive structures with yellow arrows. (**B**) Representative kymographs from confocal live-cell imaging of Ctrl#2-NSCs treated with LysoTracker and DLL1-pHrodo. LysoTracker- and pHrodo single-positive vesicular structures are indicated by red and green arrows, respectively, and double-positive structures are indicated by yellow arrows. (**C**) Immunostaining for Notch1, LAMP1, and 6xHis after incubation of Ctrl#2-NSCs with DLL1-6xHis for 30 min. Arrows indicate puncta colocalizing for all three proteins. (**D** to **F**) Western blot against cleaved N1ICD, total Notch1, and actin and respective quantifications of Ctrl#1-NSCs treated with 0.1% (v/v) DMSO, 20 μM DAPT, 100 nM BafA, or 200 μM Leu for 2 hours (*n* = 4, one-way ANOVA with Bonferroni’s multiple comparison test, means ± SEM). (**G**) Fold changes of gene expression of Notch downstream targets *HES1*, *HES5*, and *HEY1* after treatment of Ctrl#2-NSC with 0.1% (v/v) DMSO, 20 μM DAPT, 100 nM BafA, or 200 μM Leu for 2 hours (*n* = 3, one-way ANOVA with Bonferroni’s multiple comparison test, means ± SEM). Scale bars, 20 μm (A and C), 5 μm [zoomed in (C)], and 10 μm (B). Time scale, hh:minmin:ss (A). *P < 0.05. See also fig. S2.

To investigate the mechanism of receptor transport from the cell surface to lysosomes, we treated the cells with the dynamin inhibitor dynasore and found a decreased colocalization of both antigens with a significant reduction in Manders’ coefficients for Notch1-LAMP1 co-occurrence (Fig. 2, D to F). Colocalization of the Notch1 extracellular domain (N1ECD) with clathrin, but not with lipid raft–associated choleratoxin subunit B (CTB), indicates that the internalization of Notch1 receptors is mediated by clathrin (Fig. 2G). Together, these experiments speak for an internalization of Notch1 receptors via clathrin- and dynamin-mediated endocytosis and a transport of Notch1 receptors toward LAMP1^+^ lysosomes in our NSC system. To decipher whether the internalization and transport of Notch1 to acidic lysosomes is initiated upon ligand binding, we used recombinant pHrodo-labeled human delta-like protein 1 (DLL1) Notch ligand and performed an internalization assay. The pHrodo dye is fluorogenic in acidic environment of lysosomes, and the first pHrodo signal became apparent by 30 min after adding the labeled ligand, increased over time, and could be completely blocked by addition of dynasore, again indicating a dynamin-driven process (Fig. 3A and fig. S2C). The pHrodo signal colocalizes with the fluorescent signal of the LysoTracker dye, indicating colocalization in lysosomes (Fig. 3A). Kymographs based on an additional confocal live-cell imaging showed not only the internalization of DLL1-pHrodo but also the intracellular dynamics of pHrodo^+^LysoTracker^+^ vesicles. (Fig. 3B). Some pHrodo^+^ structures remain or turn LysoTracker^−^, which might be due to the fact that LysoTracker only stains a subpopulation of highly acidified lysosome, representing only a subfraction of all vesicles marked by LAMP1 (fig. S2D). To confirm the targeted internalization of the ligand upon its binding to the receptor, we performed immunostaining after 30 min of DLL1 treatment. We were able to detect internalized DLL1 punctae colocalizing with Notch1 staining, indicating that DLL1 is not randomly internalized but as a ligand-receptor complex (Fig. 3C). At 60 min of DLL1 treatment, internalized DLL1-Notch complexes were identified to segregate to the siblings during mitotic events (fig. S2E). Further evidence that these complexes are transported into lysosomes is shown by costaining of DLL1^+^Notch1^+^ punctae with LAMP1 (Fig. 3C).

As the Notch1-DLL1 complex is transported to lysosomes and the γ-secretase subunit Presenilin1 and LAMP1 showed a similar distribution along the sucrose gradient, we asked whether activation of Notch signaling is dependent on the acidifying environment within endolysosomes. To address this question, we treated NSCs with the vacuolar type H^+^ adenosine triphosphatase inhibitor bafilomycin A1 (BafA) to prevent the acidification of endolysosomes (fig. S2F). Immunoblot analysis of Notch1 intracellular domain (N1ICD), the γ-secretase cleavage product of Notch1, revealed a significant reduction in the amount of N1ICD and a slight accumulation of uncleaved Notch1 receptor (Fig. 3, D to F). Treatment of the cells with the γ-secretase inhibitor DAPT (N-[N-(3, 5-difluorophenacetyl)-l-alanyl]-s-phenylglycinet-butyl ester) showed a very similar effect, supporting the idea that the activating cleavage of Notch is dependent on the intravesicular drop in pH. In contrast, inhibition of lysosomal proteases with leupeptin (Leu) changed neither the overall Notch1 receptor levels nor the amount of N1ICD (Fig. 3, D to F). DAPT treatment for 2 hours also leads to an accumulation of Notch receptors within LAMP1^+^ vesicles (fig. S2G), indicating that prevention of γ-secretase cleavage increases uncleaved Notch1 in LAMP1^+^ vesicles. The activation of the signaling pathway was further analyzed by the transcriptional activation of the target genes, *HES1*, *HES5*, and *HEY1*. In line with the results on the protein level, the expression of Notch target genes was decreased significantly and, to a similar extent, when NSCs were treated with DAPT or BafA (Fig. 3G). Together, these data indicate that in our NSC system, internalization of Notch receptors is dependent on ligand binding, and the activating proteolytic cleavage of the receptor is dependent on the acidifying environment in endolysosomes.

### Asymmetric LysoTracker segregation is associated with an increase in HES1 expression

**Fig. 4.**
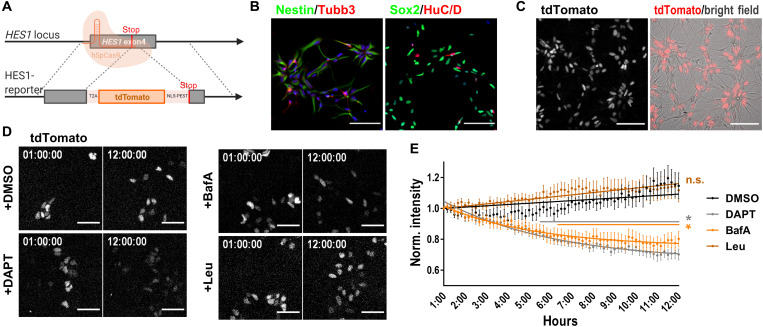
HES1 reporter tracks Notch signaling activity in NSCs. (**A**) Targeting strategy for CRISPR-Cas9–mediated generation of HES1 reporter cell line using Ctrl#2-iPSCs (for characterization see fig. S3). (**B**) Immunostaining for NSC (Nestin and Sox2) and neuronal markers (Tubb3 and HuC/HuD) in HES1 reporter NSCs. (**C**) Representative bright-field images of CRISPR-Cas9–edited rosette-type NSCs with tdTomato expression indicating active Notch signaling. (**D** and **E**) Representative frames from live-cell imaging and respective intensity sum quantification using HES1 reporter NSCs treated with 0.1% (v/v) DMSO, 20 μM DAPT, 100 nM BafA, or 200 μM Leu for up to 12 hours (*N* = 27 cells from three independent experiments, two-way ANOVA with Bonferroni’s multiple comparison test, means ± SEM). Also shown are the exponential regression curves for DAPT and BafA treatment and the linear regression for DMSO and Leu. Scale bars, 100 μm (B and C) and 50 μm (D). Nuclei were counterstained with DAPI in (B). Time scale hh:minmin:ss. *P < 0.05. See also fig. S3.

Our data so far indicate that LAMP1^+^ vesicles, which are asymmetrically inherited by dividing NSCs, also segregate Notch1 asymmetrically together with γ-secretase components to potentially activate Notch signaling by liberation of Notch ICD. To investigate whether daughter cells generated after an asymmetric distribution of lysosomes show differences in Notch activity, we generated a Notch signaling reporter cell line by introducing a modified sequence for the tdTomato fluorophore into the endogenous locus of the Notch target gene *HES1* using CRISPR-Cas9–mediated gene editing (Fig. 4A). *HES1* was linked via a T2A sequence to a nuclear localization sequence enriched in proline (P), glutamic acid (E), serine (S) and threonine (T) (NLS-PEST)-tdTomato to ensure that HES1 protein function is not disturbed. The generated HES1 (Hes family bHLH transcription factor 1) reporter line expresses the pluripotency markers Oct3/4, Sox2, and stage-specific embryonic antigen 4 (SSEA4) (fig. S3A). The scarless integration of the construct was verified by Sanger sequencing (fig. S3B). NSCs generated from targeted iPSCs show comparable morphology and marker expression as wild-type NSCs (Fig. 4B). As expected, iPSCs did not show Notch signaling activity and hence no tdTomato expression, whereas NSCs derived thereof showed high levels of HES1-driven tdTomato expression (Fig. 4C and fig. S3C). Treatment of NSCs with the Notch signaling inhibitor DAPT led to forced neuronal differentiation visible by morphological changes of the culture, which was accompanied by down-regulation of the HES1 reporter signal (fig. S3D). Inhibiting protein synthesis by cycloheximide was used to measure the half-life of the HES1 reporter, which was around 3.2 hours (fig. S3E). The HES1 reporter line was sensitive enough to monitor inhibition of Notch signaling by DAPT treatment or inhibition of vesicle acidification by BafA (Fig. 4, D and E). In contrast and as shown before when investigating protein levels of N1ICD, inhibition of lysosomal protease activity did not change HES1 reporter signal (Fig. 4, D and E).

**Fig. 5.**
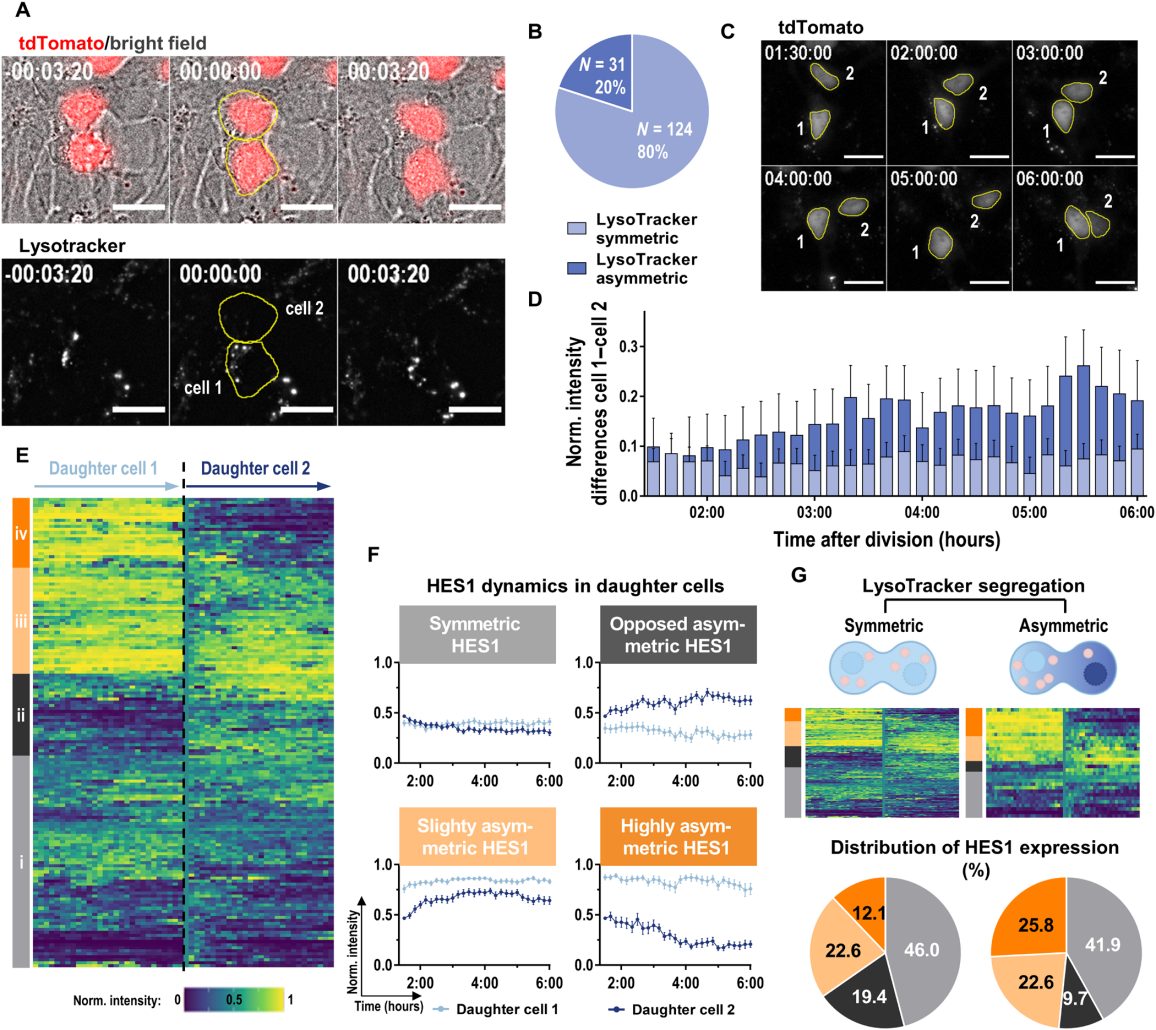
Lysosome asymmetry is correlated with differential HES1 expression in NSC-derived daughter cells. (**A**) Representative time frames from live-cell imaging of a mitotic HES1 reporter NSC stained with LysoTracker. On the basis of quantification of the LysoTracker intensity sum, daughter cell1 was defined as the cell receiving more LysoTracker signal at the end of mitosis (time point 0). (**B**) Distribution of LysoTracker symmetric and asymmetric cell division (*n* = 10 independent experiments). (**C**) Representative time frames of the tdTomato signal in daughter cells 1 and 2 starting 1.5 to 6 hours after mitosis. (**D**) Differences in *z*-normalized intensity sums between daughter cell 1 and cell 2 in LysoTracker symmetric and asymmetric divisions (*N* = 124/31 for symmetric/asymmetric cell divisions from 10 independent experiments, means + SEM). (**E**) Heatmap and clustering of HES1 expression dynamics in daughter cell pairs into four groups: (i) symmetric, (ii) opposed asymmetric, (iii) slightly, and (iv) highly asymmetric. (**F**) Dynamics of tdTomato fluorescence intensities in the different groups (*N* = 70, 27, 35, and 23 for groups 1 to 4, respectively, from 10 independent experiments, means ± SEM). (**G**) HES1 expression dynamics and cluster frequency after symmetric and asymmetric inheritance of LysoTracker during cell division of HES1 reporter NSCs (*N* = 124/31 of symmetric/asymmetric cell divisions from 10 independent experiments). 20 μm (A and C). Time scale hh:minmin:ss (A and C). See also fig. S3.

We then used the HES1 reporter cell line to monitor Notch activity in daughter cells, resulting from cell divisions with a symmetric versus asymmetric segregation of acidic vesicles. To that end, we monitored individual mitotic events of LysoTracker-stained HES1 reporter NSCs and quantified the LysoTracker signal in telophase (Fig. 5A). Identification of dividing cells and the respective daughter cells was performed on the basis of bright-field images and the tdTomato signal. Further, an asymmetry in lysosome distribution was defined analogous to the LAMP1 asymmetry in immunostaining with an index *A* > 0.2. A total of 124 symmetric and 31 asymmetric mitotic events were identified and further analyzed for the expression of the HES1 reporter in the paired daughter cells (Fig. 5B). Directly after the mitosis, both siblings show similar fluorescence intensities due to the equal inheritance of the existing tdTomato protein during mitosis. Time-lapse recordings starting at 15 min following the mitotic event show that an asymmetry of the reporter is established over the next hours (in the depicted example due to a decrease in intensity in the daughter cell that received less lysosomes; movie S1). Therefore and due to the half-life of the reporter of around 3 hours (fig. S3E), quantification of the tdTomato fluorescence dynamics was started 90 min after LysoTracker quantification, and daughter cells were followed up to 6 hours after the time point of cell division (Fig. 5C). The HES1 expression differences between daughter cells showed a clear tendency toward a biased HES1 expression of daughter cell pairs arising from a LysoTracker asymmetric cell division (Fig. 5D and fig. S3F). Because of high variations within the data, this trend was not significant. Hence, to obtain a more detailed understanding of the HES1 expression dynamics, the tdTomato patterns were clustered by their Euclidean distance into four categories [Fig. 5, E and F, and (*15*)]: (i) symmetric HES1 expression; (ii) asymmetric HES1 expression, which opposed the LysoTracker distribution during telophase; and (iii) slightly and (iv) highly asymmetric HES1 expression biased toward the daughter cell receiving more LysoTracker during cell division. When comparing HES1 reporter asymmetry in divisions showing symmetric LysoTracker distribution versus those showing asymmetric LysoTracker distribution, we observed that the percentage of symmetric and slightly asymmetric HES1-driven reporter activity did only change to a minor extent (46.0% versus 41.9% and 22.6% versus 22.6%, respectively) (Fig. 5G). In stark contrast, we observed a strong increase in the fraction of divisions, which show strong HES1 reporter asymmetry (12.1% versus 25.8%). This was accompanied by a bisection in the percentage of divisions with an opposing asymmetric reporter expression (19.7% versus 9.7%). These data support the hypothesis that a bias of Notch signaling activity in paired daughter cells is in part mediated by directed shuttling of lysosomal vesicles.

### Asymmetric segregation of lysosomes and Notch1 is associated with neurogenic cell divisions in forebrain organoids

**Fig. 6.**
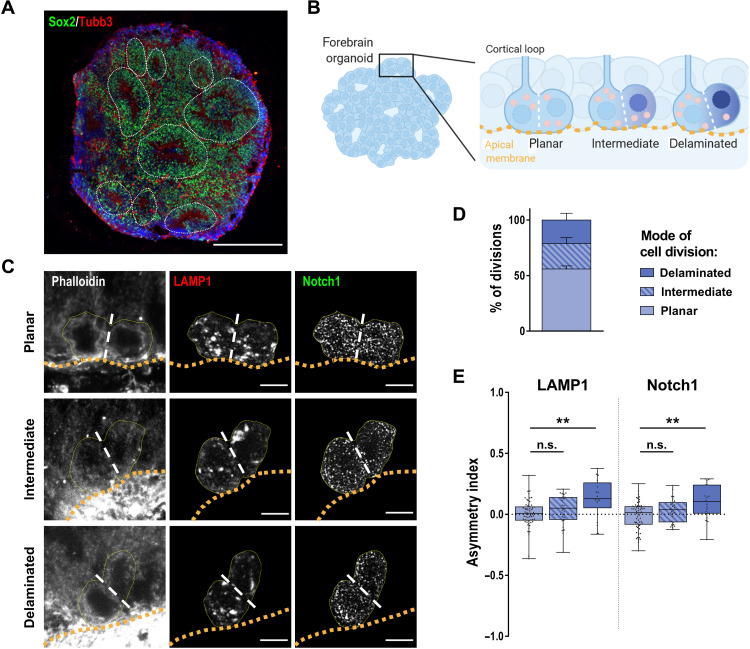
Neurogenic cell divisions in forebrain organoids show biased localization of LAMP1 and Notch1 in the apical daughter cell. (**A**) Representative image of 20-day-old forebrain organoid immunostained for NSC marker Sox2 and neuronal marker Tubb3 with cortical loops indicated by white dashed line. (**B**) Illustration of forebrain organoids with cortical loops and NSCs dividing in a planar, intermediate, or delaminating manner at the apical membrane of the loops. (**C**) Immunostaining of forebrain organoids for phalloidin, LAMP1, and Notch1. Dividing cells at the apical membrane (yellow dashed line) were identified by phalloidin staining and grouped into planar, intermediate, or delaminating cell division. LAMP1 and Notch1 signal within the area of the ROIs (yellow) is shown. (**D**) Distribution of cell division modes as defined in (B) (*n* = 3 independent experiments, means + SEM). (**E**) Quantification of LAMP1 and Notch1 asymmetry based on sum intensity ratios (see also fig. S1) between paired daughter cells (*N* = 60/28/19 of planar/intermediate/delaminating cell divisions from three independent experiments, Kruskal-Wallis test with Dunn’s multiple comparison test, box plot with dots for individual mitotic events). Scale bars, 300 μm (A) and 5 μm (C). Nuclei were counterstained with DAPI in (A). **P < 0.01.

So far, our data indicate that Notch activity is asymmetrically inherited in dividing daughters of NSCs in part by an asymmetric inheritance of Notch1-containing acidic lysosomes. One major question resulting from this observation is whether the daughter cells generated by such a division are different in their cellular identity. Therefore, we set out to investigate cell divisions in human cerebral organoids. It is consensus that in the organoid system, comparable to the mammalian cortex in vivo, symmetric and asymmetric divisions can be distinguished by the position of the dividing cells with respect to the apical lumen of cortical loops (*16*, *17*). Forebrain organoids at day 20 consist of several cortical loops organized of Sox2^+^ progenitors along a ventricular-like structure (Fig. 6A). We identified mitotic cells at the ventricular lining by phalloidin and 4′,6-diamidino-2-phenylindole (DAPI) staining and defined them as planar, delaminating, or intermediate divisions based on the attachment to the apical membrane (Fig. 6, B and C). We found 55.9 ± 2.8% of planar, 22.8 ± 5.4% of intermediate, and 21.3 ± 6.0% of delaminating mitotic events in three batches of organoids analyzed (Fig. 6D). Quantification of LAMP1 and Notch1 distribution in planar cell divisions showed mostly symmetric segregation with a mean asymmetry index of 0.004 ± 0.014 and − 0.012 ± 0.015, respectively (Fig. 6, C and E). Intermediate cell divisions already showed a tendency toward a more asymmetric distribution, and delaminating cell divisions showed a significant increase in the asymmetry indices with 0.130 ± 0.036 and 0.098 ± 0.031 for LAMP1 and Notch1, respectively (Fig. 6E). This links asymmetry of LAMP1 and Notch1 and the subsequent bias in Notch signaling activity shown above with neurogenic cell divisions in a model system for the developing human cortex. The NSCs attached to the apical membrane receive more lysosomes and hence higher Notch activity, retaining their stem cell identity, whereas cells delaminated from the apical membrane are depleted from active Notch signaling and potentially undergo neuronal differentiation after mitosis.

## DISCUSSION

Vesicular components of the endolysosomal pathway have recently been in research focus as asymmetrically inherited during mitosis (*3*–*5*). In SOPs of *D. melanogaster* and spinal cord progenitors of the neural tube of zebrafish, a subset of endosomes marked by Sara were shown to traffic Notch receptors and Notch ligands to that sibling, which continues to proliferate, indicating that this bias of Notch components is associated with the maintenance of stem cell/progenitor properties (*4*, *5*). Whereas the mechanistic basis of vesicle transport are fairly well understood in these model organisms and rely on internal cellular polarity and the transport along polarized microtubules (*4*–*6*, *18*, *19*), the mechanism of potential Notch activation remained elusive. More recently and in the mammalian hematopoietic lineage, asymmetric inheritance of lysosomes and other components of the degradation machinery was shown to allow predictions about the metabolic state of the receiving cells and about arising cellular diversity (*3*). Also in this study, Notch pathway components where demonstrated to cosegregate, although the impact of Notch activity was not addressed further.

In our study, we demonstrate that lysosomes segregate asymmetrically in human NSCs and that these lysosomes contain ligand-bound Notch receptors. We show that upon binding of recombinant ligands, the ligand-receptor complex is processed via the endocytic pathway into lysosomes and that components of the γ-secretase complex are present in the same subcellular fractions when separated in density gradients. This is in line with published studies on the subcellular localization of γ-secretase (*20*, *21*), and our findings that inhibition of lysosomal acidification biologically mimics specific γ-secretase inhibition. These data indicate a link between Notch cleavage and the acidic environment of the lysosomal compartment, suggesting a previously unidentified mechanism on how the vesicle-receiving sibling cell might bias levels of Notch activity after division. We, therefore, went one step further and determined Notch activity in the receiving cells by combined live-cell detection of asymmetric lysosomal inheritance with a Notch reporter cell line, which we generated by gene targeting. These experiments demonstrate that the asymmetry of lysosomes in dividing NSCs correlates with a Notch activity bias in the daughter cells.

Our experimental setting does not allow to follow the sibling and their progeny long term and to generate lineage relations. By that, we cannot conclude that the lysosome and hence Notch-receiving cells maintain a more proliferative, stem cell–like fate. What, however, underlines this hypothesis is the observation we made in human brain organoids. It is consensus that the plane of cell division and the degree of attachment to the apical membrane allow predictions about the cellular fates of the daughter cells, namely, that the cell that moves away from the ventricular area and shows less contact with the apical membrane is prone to differentiate, whereas the cell that remains attached to the apical membrane remains a stem cell (*16*, *17*, *22*, *23*). In our experiments, exactly these membrane-attached cells received more lysosomes and more Notch receptors, which is in line with the above-mentioned hypothesis. Sara^+^ endosomes did not show any tendency for asymmetric inheritance in our NSC system. Together with the data from the hematopoietic system, this might indicate that the cellular compartment for biased Notch inheritance has evolutionally switched from invertebrates and lower vertebrates to mammals. It is important to note that we did not address metabolic states of the receiving cells as the study performed in the hematopoietic system did not address Notch activity (*3*). It is tempting to speculate that the obvious similarities with respect to lysosomal inheritance in both lineages describe a common mechanism of human stem cells to generate siblings of different quality. It would be thus interesting to investigate other stem cell and progenitor population as well as other species to identify if and when lysosomes became center signaling hubs in human stem and progenitor cells and if, when, and why this has potentially moved from endosomes to the more acidic lysosomes.

## MATERIALS AND METHODS

### Ethical statement

iPSCs used in this study were generated from healthy donors. Donors gave written informed consent. All experiments with human material are in accordance with the Declaration of Helsinki.

### General cell culture

All cells were cultured at 5% and 37°C in the presence of 1:100 penicillin-streptomycin (Invitrogen, 15140122) and tested periodically for mycoplasma by polymerase chain reaction (PCR). Cell culture plates were coated with 1:100 Geltrex (Invitrogen, A1413302) before use, unless otherwise stated. N2 supplement was prepared in the laboratory as follows: Dulbecco’s modified Eagle’s medium (DMEM)/F12 (Invitrogen, 11320074), insulin (500 μg/ml) (Sigma-Aldrich, 91077C), transferrin (10 mg/ml) (Sigma-Aldrich T3705), sodium selenite (520 ng/ml) (Sigma-Aldrich, A8960), putrescine (1.611 mg/ml) (Sigma-Aldrich 51799), and progesterone (630 ng/ml) (Sigma-Aldrich, P8783). More details on the different cell types and their maintenance can be found in the following sections.

### iPSCs culture

iPSCs were reprogramed from dermal fibroblasts of two healthy adult donors (ctrl#1: male, age of biopsy: 33; ctrl#2: female, age of biopsy: 44) using Sendai virus. iPSCs were maintained on Geltrex-coated plates in feeder-free conditions in E8 medium: DMEM/F12 with Hepes (Invitrogen, 11330057), sodium selenite (14 ng/ml) (Sigma-Aldrich, A8960), l-ascorbic acid phosphate (64 μg/ml) (Sigma-Aldrich, A8960), insulin (20 μg/ml) (Sigma-Aldrich, 91077C), transferrin (11 μg/ml) (Sigma-Aldrich, T3705), FGF2 (100 ng/ml) (Cell Guidance Systems, GFH146), and transforming growth factor–β (2 ng/ml) (Cell Guidance Systems, GFH39). Medium was change every day. Splits were performed using EDTA (Invitrogen, 15575020), and 5 μM Y-27632 (Cell Guidance Systems, SM02) was added to the medium for 24 hours.

### Neural induction and cultivation of NSCs

Neural fate was induced in iPSCs by dual-Smad and Wnt inhibition. Therefore, medium of confluent iPSC cultures was changed to neural induction medium with 200 nM LDN193189, 500 nM A83, and 2 μM XAV 939 in DMEM/F12 (Invitrogen, 11320074), 0.5% N2 (self-made, see above), 1% B27 (Invitrogen, 17504044), 1% GlutaMAX (Invitrogen, 35050038), 1% nonessential amino acid (NEAA) (Invitrogen, 11140035), 4.44 mM glucose (Roth HN06.2). Medium was changed every day, and after 10 days, cells were split in low density with TrypLE. For further maintenance, cells were kept in DMEM/F12 medium containing 3 μM CHIR99021 and 500 nM purmorphamine in DMEM/F12 (Invitrogen, 11320074) supplemented with 0.5% N2 (self-made, see above), 0.5% B27 (Invitrogen, 17504044), 1:100 glutamine (Invitrogen, 25030024), and 4.44 mM glucose (Roth HN06.2). Medium was changed every day and every other day on the weekend. Cells were passaged using trypsin/EDTA (Invitrogen, 15400054) and trypsin inhibitor (Invitrogen, 17075029).

Before the experiments, medium was switched to DMEM/F12 (Invitrogen, 11320074) with 0.5% N2 (self-made, see above), 1% B27 (Invitrogen, 17504044), 8.88 mM glucose (Roth HN06.2), EGF (10 ng/μl) (Cell Guidance Systems, GFH26), and FGF2 (10 ng/μl) (Cell Guidance Systems, GFH28) to induce the switch from SM-NPCs to rosette-type NSCs for at least 3 days. For asymmetry quantification, NSCs were kept in EGF/FGF2 conditions until the first spontaneous differentiation occurs, split to the final format, and cultured for 3 days with or without FGF2.

### Cortical forebrain-type organoid generation

Organoids were generated as previously described with small adjustments (*24*). Briefly, low-attachment U-bottom 96-well plates were coated with 5% pluronic in phosphate-buffered saline (PBS). iPSCs were dissociated with TrypLE Express (Invitrogen, 12605028), and 6000 cells were seeded in E8 medium supplemented with 50 μM Y-27632 (Cell Guidance Systems, SM02). Medium was changed every other day. On day 5, medium was changed to organoid induction medium: DMEM/F12 (Invitrogen, 11320074), 0.5% N2 (self-made, see above), 1% B27 (Invitrogen, 17504044), 1% GlutaMAX (Invitrogen, 35050038), 1% NEAA (Invitrogen, 11140035), 4.44 mM glucose (Roth HN06.2), heparin (10 μg/ml) (Sigma-Aldrich, H3149), insulin (100 ng/ml) (Sigma-Aldrich, 91077C), 50 μM β-mercaptoethanol (Invitrogen, 31350010), 200 nM LDN193189, 500 nM A83, and 2 μM XAV 939 . Medium was changed every other day. On day 10, organoids were embedded in a 3:2 mix of Geltrex:medium according to (*25*). Hereafter, LDN193189, A83, and XAV 939 were withdrawn from the medium, organoids were cultured in Pluronic-coated 6-cm dishes on an orbital shaker, and medium was changed every 3 to 4 days. Organoids were analyzed at day 20 after embryoid body formation.

### Molecular cloning and nucleofection

For the generation of the HES1 reporter line, the homology arms corresponding to DNA sequence of the *HES1* gene surrounding the stop codon were amplified from genomic DNA and cloned into the pBluescript backbone (Addgene, #72835) using classic molecular cloning approaches. The *T2A* sequence (GAGGGCAGAGGAAGTCTTCTAACATGCGGTGACGTGGAGGAGAATCCCGGCCCT) was added in frame, followed by *tdTomato* and an *NLS-PEST* sequence. The respective sequences were amplified from Cytbow vector [a gift from the Livet laboratory and published in (*26*)] and from HES5 reporter vector [a gift from Henrique laboratory and published in (*27*)], respectively. Primers used for amplification were ordered from Integrated DNA Technologies: *HES1* 5′ homology arm, (5′-CGTGAATTCCTGGGGGTCACTGGTTTAG-3′ and 5′-GTTGCGGCCGCACTGTCGACGTTCCGCCACGGCCTC-3′), *HES1* 3′ homology arm (5′-GAGTCGACAGTACCGGTCGGGAGCTCCACCTCTCTTCCCTCCGG-3′ and 5′-GCAGCGGCCGCTTGCTTTAAGAGGGTGCG-3′), *tdTomato* (5′-TGCACCGGTATGGTGAGCAAGGGCGAG-3′ and 5′-ACGGAGCTCGTAACGCGTCTTGTACAGCTCGTCCATG-3′), and *NLS-PEST* (5′- TGCACGCGTCCTCCAAAAAAGAAGAGAAAG-3′ and 5′-TCGGAGCTCCTACACATTGATCCTAGCAG-3′). For the guide RNA, a previously published sequence was used targeting the last exon of *HES1* (*28*). Oligonucleotides were purchased from Integrated DNA Technologies and cloned into pSpCas9(BB)-2A-Puro (PX459) v2.0 (Addgene, #62988) following the distributor’s protocol.

Nucleofection of iPSCs was performed with the Nucleofector 2b and the Cell Line Nucleofector Kit V (Lonza, VCA-1003) according to the manufacturer’s protocol. Briefly, Ctrl#2-iPSCs were treated with TrypLE Express (Invitrogen, 12605028) and counted, and 106 cells were centrifuged at 300*g* for 5 min. The pellet was resuspended in 82 μl of Nucleofector solution V, 18 μl of supplement 1, and 1 μg of each plasmid. The program used for nucleofection was B-027. Cells were plated in E8 medium without penicillin-streptomycin and with 5 μM Y-27632 (Cell Guidance Systems, SM02). One day after nucleofection, puromycin (0.33 μg/ml) (EMD Millipore, #540222) was added to the media. After another 24 hours, medium was changed to E8 medium with penicillin-streptomycin, puromycin, and Y-27632. At days 3 and 4 after nucleofection, Y-27632 and puromycin were removed from the medium, respectively. Clones were picked manually into a 48-well plate once the colonies reached an appropriate size. Clones were screened by genotyping, and the integration of the reporter was verified by Sanger sequencing (Microsynth Seqlab GmbH).

### Immunofluorescence staining

For immunofluorescence staining, NSCs were split onto glass coverslips pretreated with hydrochloric acid and coated with poly-l-lysin (PLL) (100 μg/ml; Sigma-Aldrich, P2636) in 25 mM boric acid (pH 8.4) and laminin (2.5 μg/ml) (Invitrogen, 23017015). For labeling of lipid rafts, NSCs were additionally treated with CTB (10 μg/ml) for 1 hour at 4°C before fixation. Cells were fixed for 10 min in 4% paraformaldehyde (PFA) and washed with PBS, and PFA reaction was quenched for 10 min in 25 mM glycine in PBS. After another wash in PBS, the cells were blocked and permeabilized in 10% fetal bovine serum (Invitrogen, 10270106) in PBS with either 0.1% saponin or 0.3% Triton X-100 for 1 hour. Primary antibodies were incubated in blocking solution overnight at 4°C. The following primary antibodies were used with Triton X-100 permeabilization: HuC/D (1:500; Thermo Fisher Scientific, A-21271), Sox2 (1:500; Cell Signaling Technology, 3579), Tubb3 (1:1000; Synaptic Systems, 302 304), Nestin (1:600; R&D Systems, MAB-1259), Oct3/4 (1:600; Santa Cruz Biotechnology, sc5279), and SSEA4 [1:600; Developmental Studies Hybridoma Bank (DSHB), MC-813-70). Saponin permeabilization was used for the following primary antibodies: LAMP1 (1:400; DSHB, H4A3), CD63 (1:400; ExBio Praha, 11-343-C100), EEA1 (1:100; Cell Signaling Technology, 48453), LC3 (1:100; Cell Signaling Technology, 12741), Rab5 (1:100; Cell Signaling Technology, 46449), Rab7 (1:100; Cell Signaling Technology, 9367), Rab11 (1:100; Cell Signaling Technology, 5589), Sara/ZFYVE9 (1:100; Sigma-Aldrich, HPA065852), Notch1 (1:200; Cell Signaling Technology, 4380), Notch1 (1:100, DSHB bTAN 20-c), Notch1ECD (1:50; BioLegend, 819101); Notch2 (1:400; Cell Signaling Technology, 4530), His-tag (1:500; Biotrend, CHIS-45A-Z). No permeabilization was used for the extracellular staining of Notch1ECD (1:50; BioLegend, 819101). Coverslips were washed three times with PBS or saponin blocking solution for 10 min, respectively. Secondary antibodies were diluted in respective blocking solution and applied for 1 hour at room temperature (RT) (1:1000; all Invitrogen). Where appropriate, ATTO 565 phalloidin (1:2000; Atto Tec AD, 565-81) was added to the mix. After two washing steps in PBS for 10 min, DNA was counterstained with 300 nM DAPI for 10 min at RT. After two brief washes in PBS and water, coverslips were mounted in Mowiol.

Organoids were fixed on day 20 after EB generation in 4% PFA for 10 min at RT, washed three times in PBS, and transferred to 30% sucrose in PBS to dehydrate overnight at 4°C. After embedding in 10% (w/v) sucrose and 7.5% (w/v) gelatin in PBS, organoids were cut at a cryostat in 20-μm-thick sections, which were stored at −20°C. After thawing organoid sections, they were processed according to the above protocol using 0.3% Triton X-100 permeabilization.

### Asymmetry quantification

Quantification of asymmetric protein segregation during cell division was performed on *z*-stack images of individual mitotic events. Two-dimensional (2D) cultures of NSC were therefore imaged with a Leica DM6 B microscope equipped with a DFC9000 GT camera and a HC PL APO 40×/0.95 dry objective (all Leica). 3D organoid slices were imaged at a TCS SP5 II confocal laser scanning microscope using a HCX PL APO 63×/1.40 to 0.60 oil objective (both Leica). *Z*-stacks with *z* increments of 0.29 and 0.5 μm were obtained for 2D and 3D cultures, respectively. Regions of interest (ROIs) for daughter cells were set on the basis of the DAPI and phalloidin signal, and the mean signal intensity for each daughter cell was measured on sum intensity projections of *z*-stacks. Background was subtracted based on secondary antibody control staining, and intensity sums were calculated for individual daughter cells by multiplying mean signal intensity by cell area. Asymmetry index *A* was calculated as follows

$$A=\frac{\text{Intensity}\;\sum \;\text{Cell}\;1-\text{Intensity}\;\sum \;\text{Cell}\;2}{\text{Intensity}\;\sum \;\text{Cell}\;1+\text{Intensity}\;\sum \;\text{Cell}\;2}$$

In 2D, the daughter cell with the higher intensity sum for the LAMP1/CD63 signal was set as cell 1. Asymmetry indices, therefore, range from 0, meaning completely symmetric, to 1, meaning completely asymmetric protein distribution. Asymmetry indices for Notch1 range from −1 to 1, whereby positive values indicate segregation in the same cell as LAMP1 and negative values indicate segregation to the opposing daughter cell. In 3D, cell 1 was defined as the more apical daughter cell based on the attachment to the apical membrane. In planar cell divisions, the allocation of cells 1 and 2 was randomized. To analyze asymmetric segregation of vesicle numbers, the ComDet v0.4.1 plugin for ImageJ (*29*) was used, and the asymmetry indices were calculated analogous to the formular above.

### Ligand internalization

For live-cell ligand internalization, recombinant DLL1-6xHis (R&D Systems, 1818-DL-050) was labeled with pHrodo iFL Green (Thermo Fisher Scientific, P36015) according to the manufacturer’s protocol. Briefly, 50 μg of recombinant protein was resuspended in 100 μl of ddH_2_O, and 10 μl of 1 M sodium bicarbonate was added. After addition of 6.1 μl of 2 mM pHrodo solution, the mixture was incubated for 15 min at RT, and the labeled protein was purified with the supplied columns. NSCs were grown on PLL-laminin–coated ibidi plates (for coating, see the “Immunofluorescence staining” section). Before imaging, cells were equilibrated in imaging buffer [140 mM NaCl, 2.5 mM KCl, 1.8 mM CaCl2, 1 mM MgCl2, 20 mM Hepes, and 10 mM glucose (pH 7.4)] for 30 min. During equilibration, 0.1 nM LysoTracker Deep Red, 50 μM dynasore, or 0.1% (v/v) dimethyl sulfoxide (DMSO) was added to the buffer. Then, pHrodo-labeled DLL1-6xHis was added directly to the imaging buffer in a 1:5 ratio, and imaging was started 5 min after ligand addition. Internalization was imaged with Celldiscoverer 7 microscope equipped with an Axiocam 506 (both Carl Zeiss), where cells were kept at 37°C and 5% CO_2_ during the whole-image acquisition. Plan-Apochromat 50×/1.2 water autocorr objective (Carl Zeiss) was used, and one 7-μm *z*-stack with 0.5-μm increments was taken every 2.5 min up to 1 hour after DLL1 addition. For kymographs, additional confocal live-cell imaging was performed using a TCS SP5 II microscope and a 63×/1.40 oil objective (Leica), where the object table was kept at 37°C during imaging, and cells were imaged every 10 s for 1 hour starting directly after adding the labeled ligand.

For immunostaining, human recombinant DLL1-6xHis (Sino Biological, 11635-H08H) was diluted to a concentration of 10 μg/ml in medium and added to the NSCs grown on coverslips (for coating, see the “Immunofluorescence staining” section). Cells were further incubated for 30 min at 37°C and 5% CO_2_ to allow internalization of the ligand. To remove ligand remaining on the cell surface, cells were then washed with ice-cold acetic acid buffer [200 mM acetic acid and 500 mM NaCl (pH 2.0)] for 5 min on ice. Cells were fixed in 4% PFA and processed for immunofluorescence. Representative images were acquired at a TCS SP5 II confocal laser scanning microscope using a HCX PL APO 63×/1.40 to 0.60 oil objective (both Leica).

### Manders’ co-occurrence analysis

For analysis of receptor endocytosis, NSCs were treated with either 50 μM dynasore or 0.1% (v/v) DMSO for 1 hour before fixation and subjected to immunofluorescence staining (see above). *Z*-stacks with an increment of 0.29 μm were acquired using Leica DM6 B microscope with a DFC9000 GT camera and an HC PL APO 40×/0.95 dry objective (all Leica). Background fluorescence was reduced by the small-volume computational clearing algorithm implemented in the Leica Application Suite X software (Leica). The analysis was performed on maximum intensity projections for a total of 60 cells on 10 images taken for each condition and experiment using the ImageJ plugin EzColocalization (*30*). Thresholds for LAMP1 and Notch1 were set on the basis of the top 10 and 20% of pixels, respectively. ROIs were set manually around the cytoplasm of the cells, excluding the nucleus from the analysis. Results from three independent experiments were pooled.

### HES1 reporter stability

To determine the half-life of tdTomato, HES1 reporter NSCs were treated with 100 μM cycloheximide. Imaging was started directly after cycloheximide addition, and cells were observed for 10 hours every 10 min. For the analysis of the effect of lysosomal acidification on Notch signaling, NSCs derived from the HES1 reporter line were mixed in a 1:10 ratio with wild-type Ctrl#2-NSCs. A 0.1% DMSO, 20 μM DAPT, 100 nM BafA, or 200 μM Leu was added directly before starting the imaging. The cells were tracked over a period of 12 hours every 10 min.

For both experiments, the Celldiscoverer 7 microscope with an Axiocam 506 and a Plan-Apochromat 20×/0.7 autocorr objective (all Carl Zeiss) was used, and cells were maintained at 37°C and 5% CO_2_. ROIs were set manually in ImageJ around the nucleus in every frame, background was subtracted, and the intensity sum of individual nuclei was analyzed. Exponential fitting curves were calculated in Prism 6 (GraphPad).

### Tracking mitotic events and respective daughter cells

For imaging of lysosome asymmetries and tracking of HES1 expression in the daughter cells, HES1 reporter NSCs were mixed 1:10 with wild-type Ctrl#2-NSCs and seeded on a PLL-laminin–coated 3.5-cm ibidi dish. Cells were cultured for 3 days and synchronized on the days of the experiment with 75 nM nocodazol. After 4 hours, cells were washed extensively, and fresh medium containing 0.1 nM LysoTracker Deep Red was added. Thirty minutes after washing, live-cell imaging with the Celldiscoverer 7 microscope equipped with an Axiocam 506 was started. In the first 1.5 hours, mitotic events were imaged with a Plan-Apochromat 50×/1.2 water autocorr objective (Carl Zeiss) every 200 s as 7-μm *z*-stacks with 0.5-μm increments. Afterward, the daughter cells were tracked for 14 hours with 10 min between frames with a Plan-Apochromat 20×/0.95 autocorr objective (Carl Zeiss).

The LysoTracker signal during live-cell imaging was treated similar as the sum intensity values from the immunostaining, asymmetry indices were calculated accordingly, and cell1 was defined as the daughter cell with the higher LysoTracker signal (see also Asymmetry quantification section). HES1 expression was tracked as the intensity of tdTomato signal, which was quantified as described in the chapter on HES1 reporter stability. Further, tdTomato intensity was *z*-normalized within each experiment, individual time series were normalized by setting the first time point of the cell2 to 0, and normalized data were rescaled on the basis of their percentage rank. Differences in fluorescence intensity within daughter cell pairs were calculated from normalized and ranked data for each time point as well as from raw data using the following formula

$$\frac{\text{Intensity}\;\sum \;\text{Cell}\;1(t)-\text{Intensity}\;\sum \;\text{Cell}\;2(t)}{\text{Intensity}\;\sum \;\text{Cell}\;2(t)}$$

All these analyses were performed in Excel (Microsoft). For clustering, time series of the two daughter cells were concatenated and analyzed using the time course inspector package in RStudio (*15*). Clustering was based on the Euclidean distance of the complete time series. Cluster number was set to 6 by visual inspection of the mean values of each cluster. Clusters were grouped to represent symmetric, opposed asymmetric, slightly asymmetric, and highly asymmetric HES1 expression patterns.

### Sucrose gradient

NSCs from 2 × 10–cm dishes were harvested and lysed using hypotonic shock as previously described (*14*). Briefly, cells were washed in PBS with 0.5× protease inhibitor (Thermo Fisher Scientific, A32955), scraped from the dish, and centrifuged at 200*g* for 5 min. The pellet was briefly washed with hypotonic buffer [3 mM imidazole (pH 7.4), 1 mM EDTA, and 1× protease inhibitor] and centrifuged at 200*g* and 4°C for 10 min. Then, the pellet was resuspended and kept on ice in hypotonic conditions for 15 min. Sucrose buffer [500 mM sucrose, 3 mM imidazole (pH 7.4), 1 mM EDTA, 0.06 mM cycloheximide, and 1× protease inhibitor] was added, and cells were lysed by passaging through a 22-gauge needle 10 times. Nuclei were pelleted by centrifugation at 2000*g* and 4°C for 10 min, and postnucleus supernatant was transferred to a 10 to 50% continuous sucrose gradient. Intracellular vesicles were separated by ultracentrifugation for 16 hours at 200,000*g* and 4°C. Twenty fractions, each of 500 μl, were collected, and 40 μl of each fraction was analyzed by Western blot.

### Western blot

For Notch cleavage analysis, NSCs were cultured for 2 hours in the presence of 0.1% (v/v) DMSO (Sigma-Aldrich, D5879), 20 μM DAPT (Cell Guidance Systems, SM15), 100 nM BafA (VWR International, J61835.MX), or 200 μM Leu (SERVA Electrophoresis GmbH, 51867.02); washed with PBS; and lysed in radioimmunoprecipitation assay buffer [50 mM tris-HCl (pH 7.4), 150 mM NaCl, 0.2% Triton X-100, 25 mM EDTA, 0.2% SDS, and 1× protease inhibitor] for 1 hour on ice. Samples were sonicated using 20% duty cycles, 50% output, and seven pulses and centrifuged at 16,000*g* and 4°C for 15 min. Protein concentration in the supernatant was determined by Pierce BCA Protein Assay (Thermo Fisher Scientific, 23227).

Twenty micrograms of protein lysates or 40 μl of sucrose gradient fractions was diluted in 6× protein sample buffer [375 mM tris (pH 6.8), 6% SDS, 6% glycerol, 9% β-mercaptoethanol, and 0.03% bromophenol blue] and incubated for 5 min at 95°C. Sucrose gradient fractions analyzed for CD63 were treated under nonreducing conditions, i.e., using 6× sample buffer without β-mercaptoethanol. Proteins were separated using tris-tricine SDS–polyacrylamide gel electrophoresis for 2 to 3 hours at 110 V and transferred to 0.2-μm nitrocellulose membranes using the Trans-Blot TurboTM Transfer System (Bio-Rad) for 45 min at 1 A. Membranes were blocked with 5% milk powder in tris-buffered saline with Tween (TBS-T) for 1 hour. Primary antibodies were diluted in blocking solution and incubated overnight at 4°C. Membranes were washed three times in TBS-T, and secondary antibodies were incubated for 1 hour at RT in TBS-T. Membranes were washed again three times with TBS-T and imaged on an Odyssey imager (Li-COR). The following primary antibodies were used: Notch1 (1:1000; Cell Signaling Technology, 4380), Notch1 cleaved (1:1000; Cell Signaling Technology, 4147), Presenillin1 (1:1000; BioLegend, 823404), EEA1 (1:1000; Cell Signaling Technology, 3288), Sara/ZFYVE9 (1:1000; Sigma-Aldrich, HPA065852), LAMP1 (1:1000; Cell Signaling Technology, 9091), CD63 (1:200; ExBio Praha, 11-343-C100), and actin (1:10,000; Cell Signaling Technology, 3700). The secondary antibodies used were anti-rabbit DyeLight800, anti-mouse DyeLight680, and anti-mouse DyeLight800 (all 1:15,000; all Cell Signaling Technology, 5151, 5470, and 5257).

The analysis of Notch cleavage was performed on one membrane by stripping and reprobing. Notch1 cleaved antibody was removed by repeated incubation with stripping buffer [25 mM glycine (pH 2.0)] for 5 and 40 min. Stripped membranes were washed three times with TBS-T, and the staining procedure was repeated with Notch1 antibody starting with membrane blocking.

Quantification was carried out using densiometry analysis in ImageJ. Therefore, ROIs were set around each lane, and signal intensity over background was analyzed. For the analysis of Notch cleavage, signal for cleaved Notch1 was normalized to the respective total Notch1 signal, and total Notch1 level was normalized to actin. For the analysis of the sucrose gradient, the fractions of each protein were normalized to the respective fraction with the highest protein content. Three to four biological replicates were analyzed for each experiment.

### Real-time PCR and qPCR

NSCs were harvested in PEQGOLD TriFast (VWR), and RNA was isolated according to the manufacturer’s protocol. Briefly, chloroform was added after cells were lysed, and mixture was incubated for 15 min at RT. Centrifugation at 12,000*g* for 5 min leads to separation of the different phases, and isopropanol was added to the watery phase. RNA was precipitated overnight at −20°C and spun down at 12,000*g* for 15 min. After two washing steps with 75% ethanol, RNA pellets were air-dried and resuspended in diethyl pyrocarbonate–treated water. Contaminations with genomic DNA were removed by treatment with deoxyribonuclease I (Sigma-Aldrich, AMPD1). An iScript complementary DNA (cDNA) synthesis kit (Bio-Rad, 1708891BUN) was used according to the manufacturer’s protocol to generate cDNA. Expression of Notch target genes was quantified in NSCs after 2 hours of 10 μM DAPT, 100 nM BafA, or 0.1% (v/v) DMSO treatment by quantitative PCR (qPCR). The following primers were used (all Integrated DNA Technologies): *glyceraldehyde-3-phosphate dehydrogenase (GAPDH)* (5′-TCGGAGTCAACGGATTTGGT-3′ and 5′-TGAAGGGGTCATTGATGGCA-3′), *HES1* (5′-AAAAATTCCTCGTCCCCGGT-3′ and 5′-GGCTTTGATGACTTTCTGTGCT-3), *HES5* (5′-TGAAGCACAGCAAAGCCTTC-3′ and 5′-GCAGGCACCACGAGTAGC-3′), and *HEY1* (5′-TAATTGAGAAGCGCCGACGA-3′ and 5′-GCTTAGCAGATCCCTGCTTCT-3′). Analysis was performed using QuantStudio 7 Flex (Thermo Fisher Scientific) and the GoTaq DNA Polymerase (Promega M780B). ΔΔ*C*_t_ method was applied, and expression of genes of interest was normalized to *GAPDH* expression (*31*).

### Statistics and visualization

Statistical analyses were performed in Prism 6 (GraphPad) with significance levels set as follows: *P < 0.05, **P < 0.005, and ***P < 0.001; not significant (n.s.), *P* > 0.05. Quantifications were performed on at least three biological replicates as indicated in each figure legend, and results are shown as means ± SEM. One-sample *t* test, one-/two-way analysis of variance (ANOVA), or Kruskal-Wallis test was applied together with Bonferroni’s or Dunn’s multiple comparison test as indicated in each figure legend. Graphs were generated with Prism 6 (GraphPad), and schematic representations in Figs. 4 and 5 and fig. S1 were generated with the help of biorender.com.

## Notes

View/request a protocol for this paper from *Bio-protocol*.
