# Supplementary material for: Asymmetric Notch activity by differential inheritance of lysosomes in human neural stem cells
Source: Sci Adv. 2022 Feb 11;8(6):eabl5792. doi: 10.1126/sciadv.abl5792 (PMC8836802; doi:10.1126/sciadv.abl5792)
Supplement: Supplementary file 1 — Figs. S1 to S3 [file sciadv.abl5792_sm.pdf]

Science Advances
AAAS

Supplementary Materials for

Asymmetric Notch activity by differential inheritance of lysosomes in human neural stem cells

Bettina Bohl, Ammar Jabali, Julia Ladewig, Philipp Koch*

*Corresponding author. Email: philipp.koch@zi-mannheim.de

Published 11 February 2022, *Sci. Adv.* **8**, eabl5792 (2022)
DOI: 10.1126/sciadv.abl5792

The PDF file includes:

Figs. S1 to S3

Other Supplementary Material for this manuscript includes the following:

Movie S1

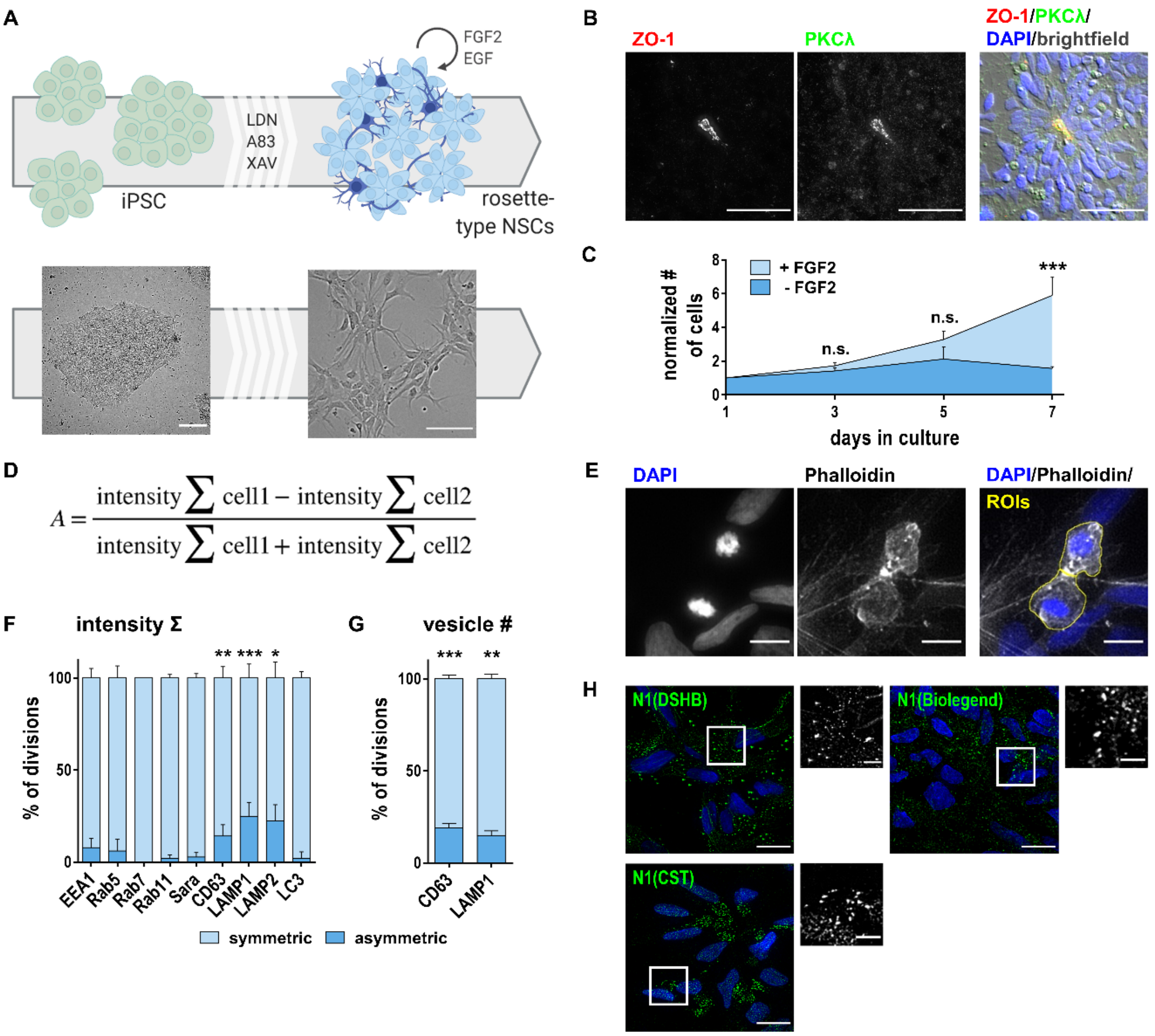

$$A = \frac{\text{intensity}\sum \text{cell1} - \text{intensity}\sum \text{cell2}}{\text{intensity}\sum \text{cell1} + \text{intensity}\sum \text{cell2}}$$

Fig. S1.

Derivation of NSCs from human iPSCs and analysis of asymmetric cell division. (**A**) Differentiation protocol from human iPSCs to rosette-type NSCs and representative brightfield images of the two cell types. (**B**) Immunostaining for tight junctions (ZO-1) and cellular polarity factors (PKCλ) in Ctrl#1 rosette-type NSCs. Brightfield image and DAPI staining indicate rosette morphology of NSCs. (**C**) Quantification of total cell number based on DAPI staining of Ctrl#1-NSCs within 10 representative images for each time point and condition (n = 3, two-way ANOVA with Bonferroni's multiple comparison test, mean + SEM). (**D-E**) Calculation of the asymmetry index A using the intensity sum within the ROIs for daughter cell1 and daughter cell2. Strategy to manually set ROIs for the two daughter cells during telophase of mitosis based on DAPI and Phalloidin staining. (**F-G**) Quantification of asymmetric distribution of vesicle markers between paired daughter cells during mitosis based on sum intensity ratios (F) and vesicle number ratios (G) (N = 3-6 coverslips from 3 independent experiments with n=39-58 cells per coverslip, one-sample t-test, mean + SEM). (**H**) Immunostaining for Notch1 receptors in Ctrl#1-NSCs with different antibodies against Notch1: #4380, Cell Signaling Technology (CST); #bTAN 20-c, Developmental Studies Hybridoma Bank (DSHB); #819101, Biolegend. Scale bars, 100 µm (A),

50 µm (B), 10 µm (E), 20 µm (H), 5 µm (zoom in H). Nuclei were counterstained with DAPI. *$p<0.05$, **$p<0.01$, ***$p<0.001$, n.s. - not significant.

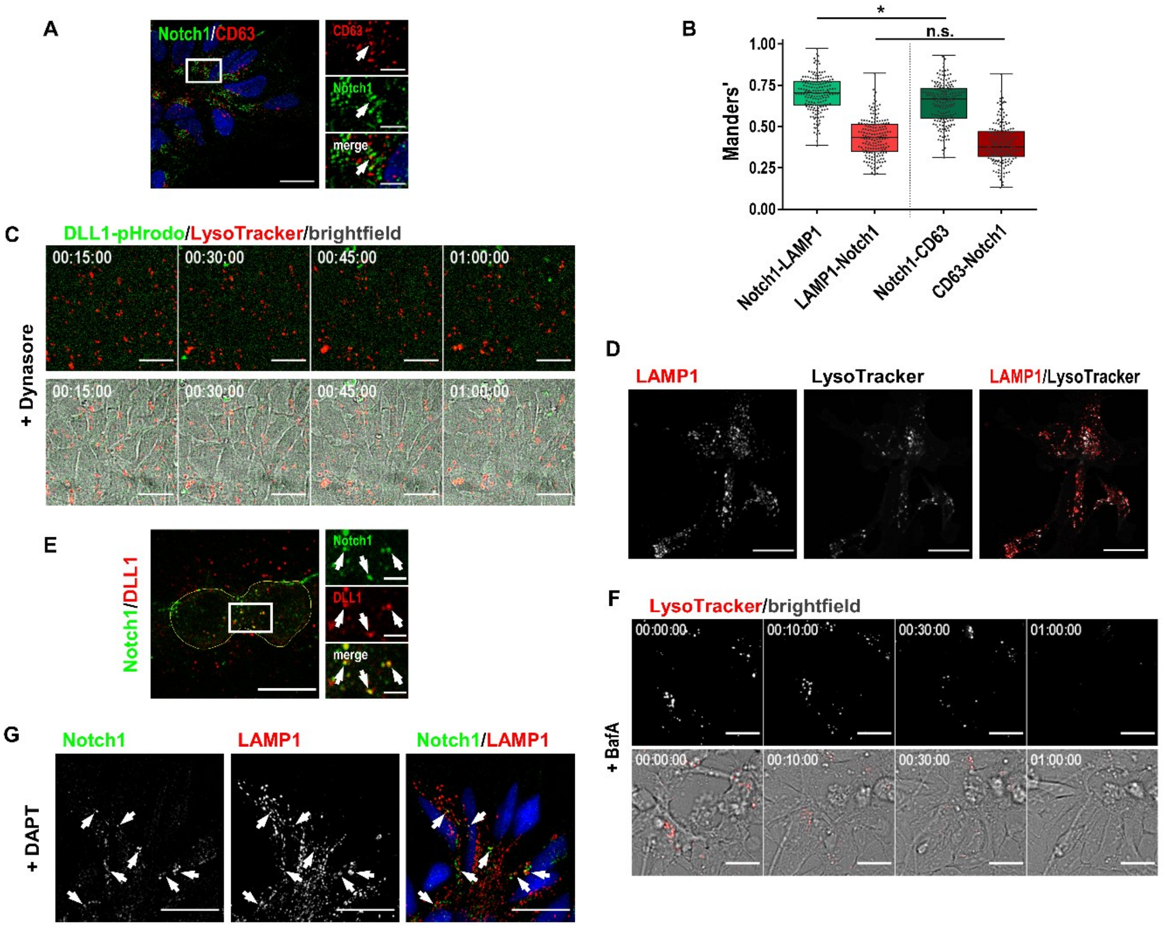

Fig. S2.

Inhibition of Notch1 receptor internalization and activation. (**A**) Immunostaining for Notch1 and CD63 in Ctrl#2-NSCs. (**B**) Manders' co-occurrence analysis of Notch1 receptors with LAMP1 (as also shown in Fig. 2D,F) and CD63, respectively (N = 179 cells from 3 independent experiments, Kruskal-Wallis test with Dunn's multiple comparison test, box plot with dots for individual cells). (**C**) Ctrl#2-NSCs were pretreated with 50 µM Dynasore while stained with LysoTracker. Representative frames from live cell imaging after addition of DLL1-pHrodo at time point 0. (**D**) Immunostaining for LAMP1 of Ctrl#1-NSCs stained with 100 nM LysoTracker for 30 min before fixation. (**E**) Immunostaining in a mitotic cell for Notch1 and 6xHis after incubation of Ctrl#1-NSCs with DLL1-6xHis for 60 min. Arrows indicate punctae of Notch1-DLL1-complexes in vesicular structures. (**F**) Representative time frames of LysoTracker-stained Ctrl#1-NSCs directly after starting a treatment with 100 nM BafA. (**G**) Immunostaining of Ctrl#1-NSCs after 2 h treatment with 20 µM DAPT for Notch1 and LAMP1 showing an increased accumulation of Notch1 receptors in LAMP1^{+} vesicles (white arrows). Scale bars, 20 µm (A,C,D,F,G), 10 µm (E), 5 µm (zoom in A), 2 µm (zoom in E). Nuclei were counterstained with DAPI in (A,G). Time scale, hh:minmin:secsec. *$p<0.05$, n.s. - not significant.

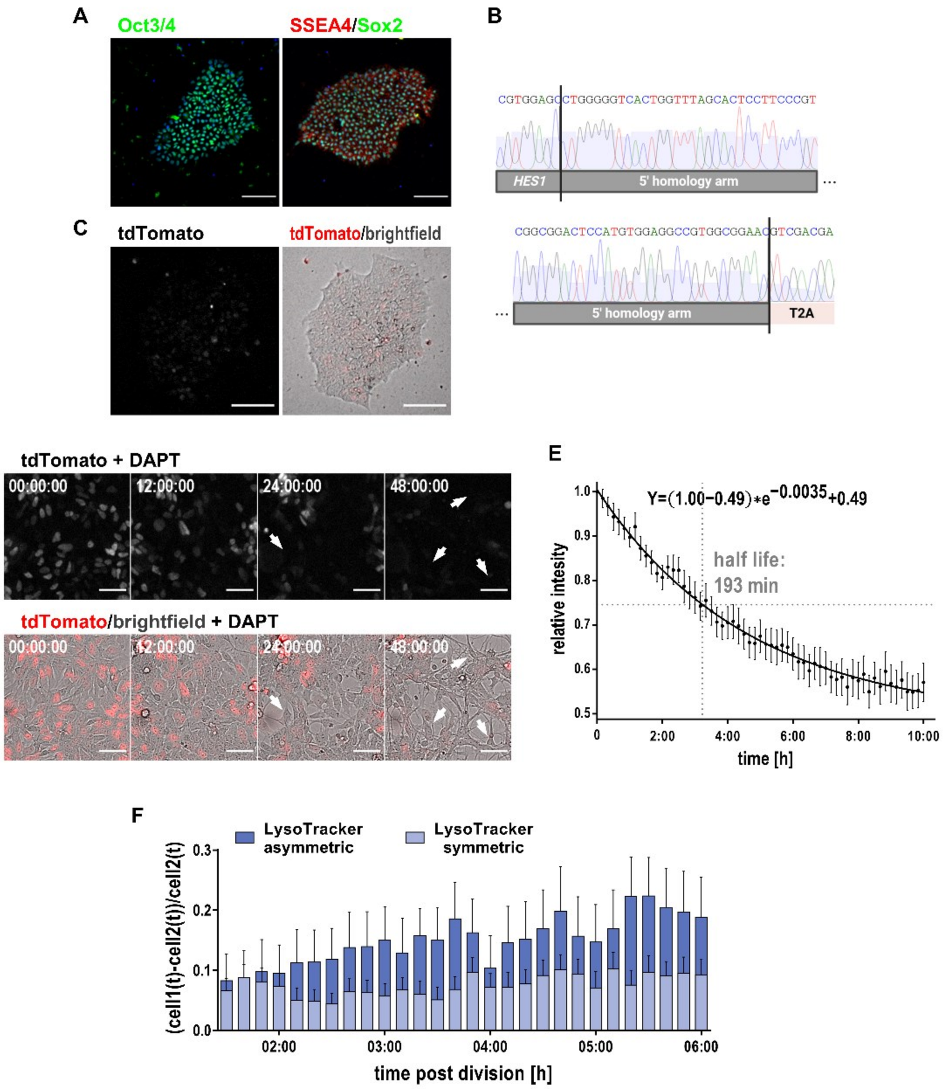

Fig. S3.

Characterization of HES1-reporter line. (**A**) Immunostaining for pluripotency markers Oct3/4, SSEA1 and Sox2 in CRISPR/Cas9-edited Ctrl#2-iPSCs (HES1-reporter line). (**B**) Sanger sequencing of the edited *HES1* locus indicating scarless integration of the reporter construct. (**C**) Representative brightfield images of HES1-reporter iPSCs showing only weak Notch signaling activity in pluripotent cells. (**D**) Representative frames from live cell imaging of HES1-reporter NSCs treated with 10 µM DAPT for 48 h. White arrows indicate cells with low tdTomato signal and neuronal morphology. (**E**) Half-life of tdTomato was calculated by cycloheximide chase (100 µM) and tracking of the tdTomato sum intensity in individual cells for 10 h (N = 22 cells from 3 independent experiments, mean ± SEM). Also shown is the exponential regression curve and the respective formular. (**F**) Alternative calculation of daughter cell differences by normalization to signal intensity of cell2 at each time point (compare to Fig. 4I; N = 124/31 for symmetric/asymmetric cell divisions from 10 independent experiments, mean + SEM). Scale bars, 100 µm (A,C), 50 µm (D). Nuclei were counterstained with DAPI. Time scale, hh:minmin:secsec.
